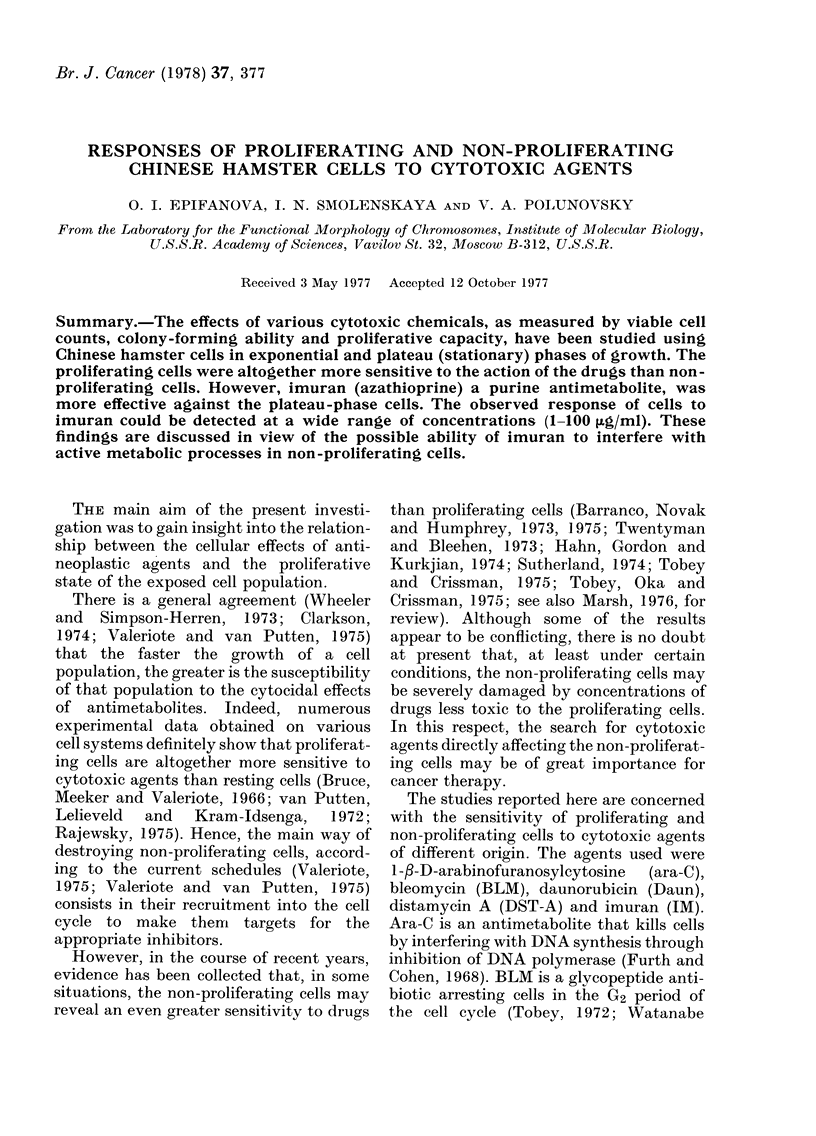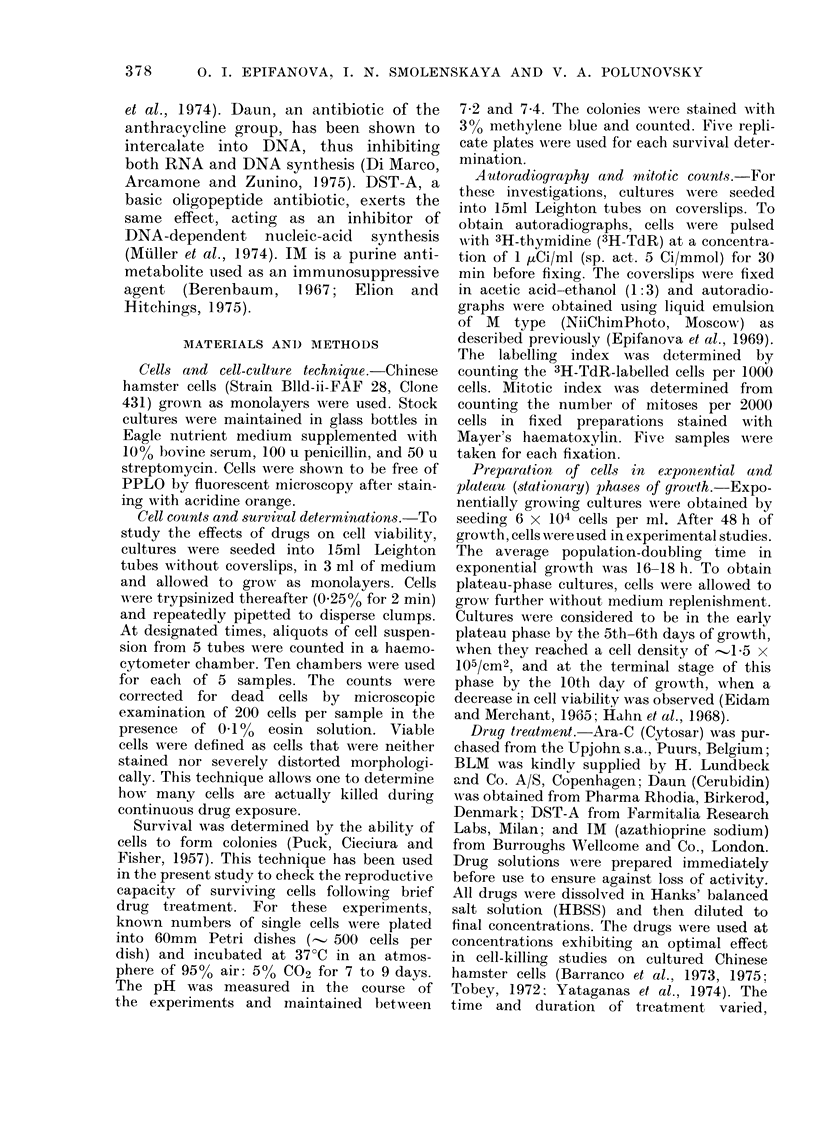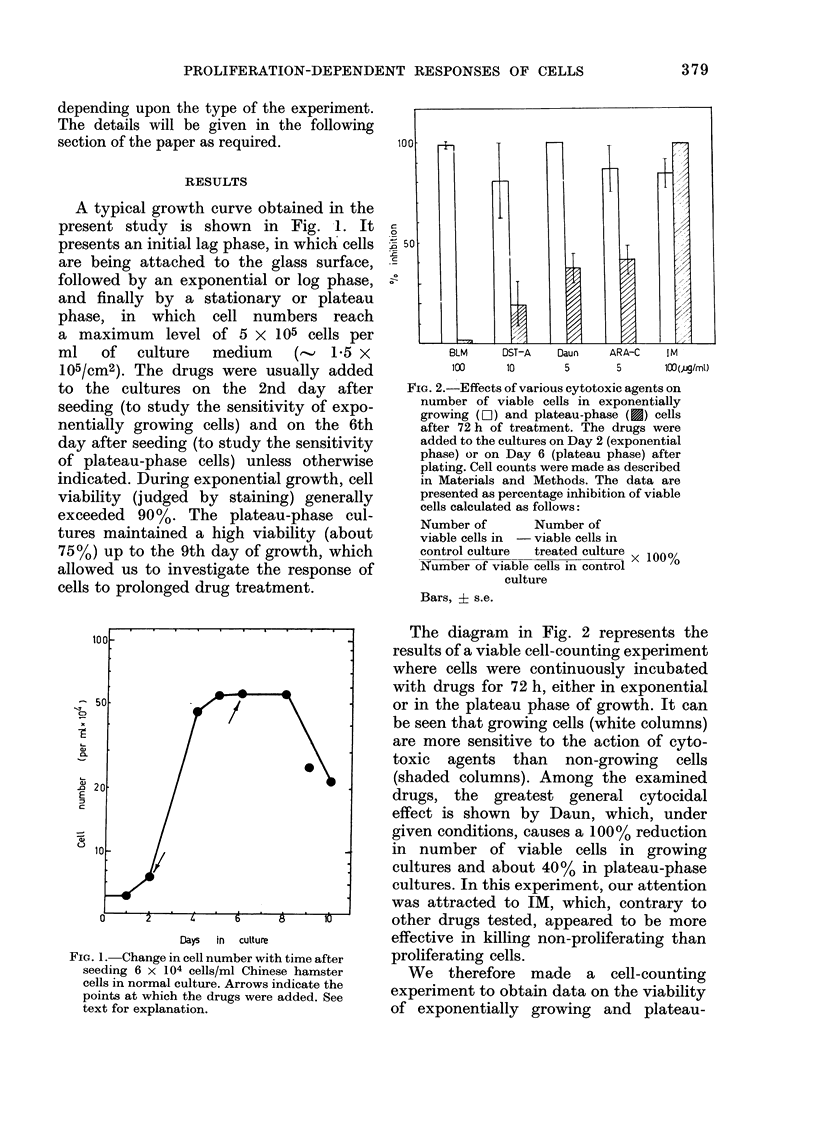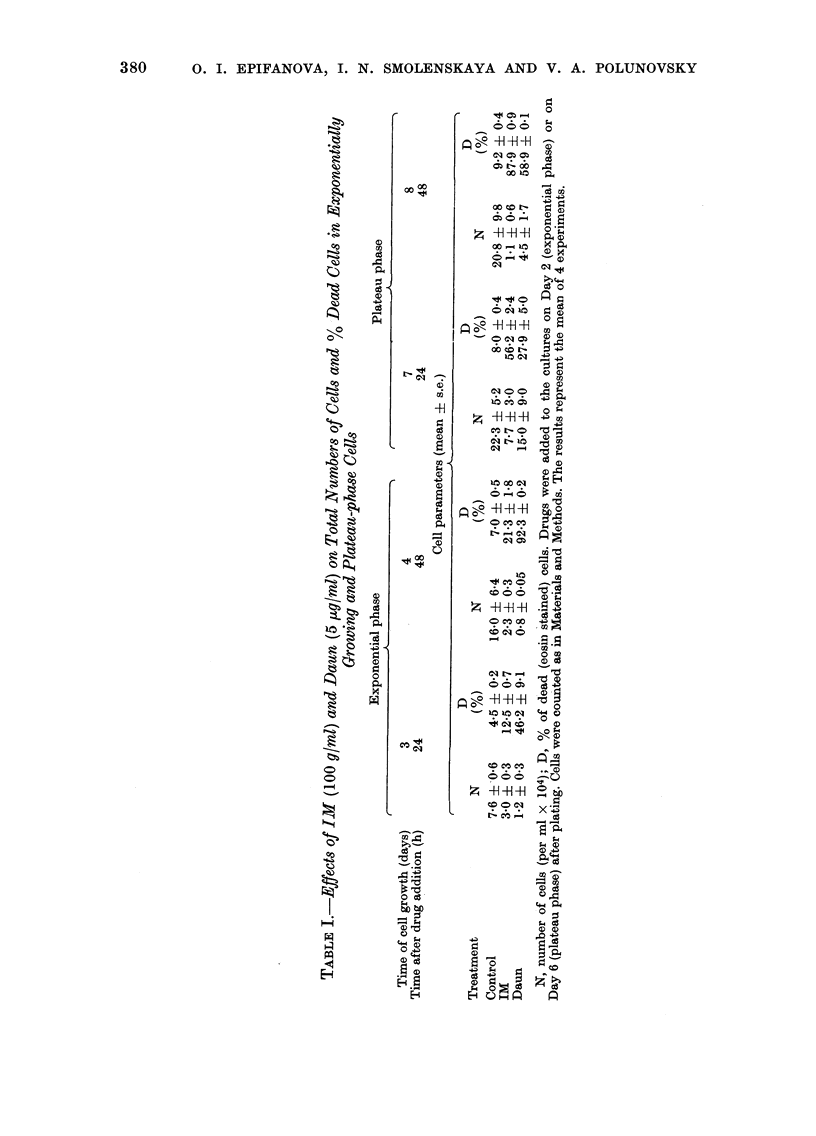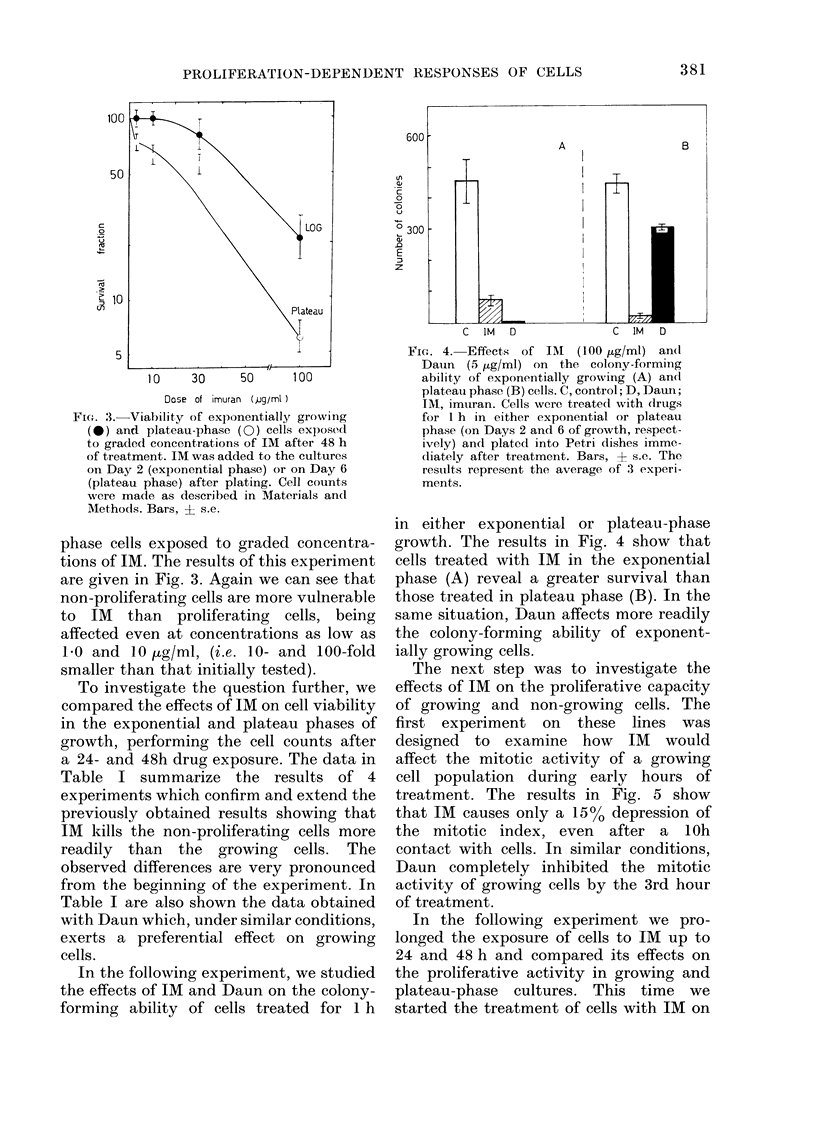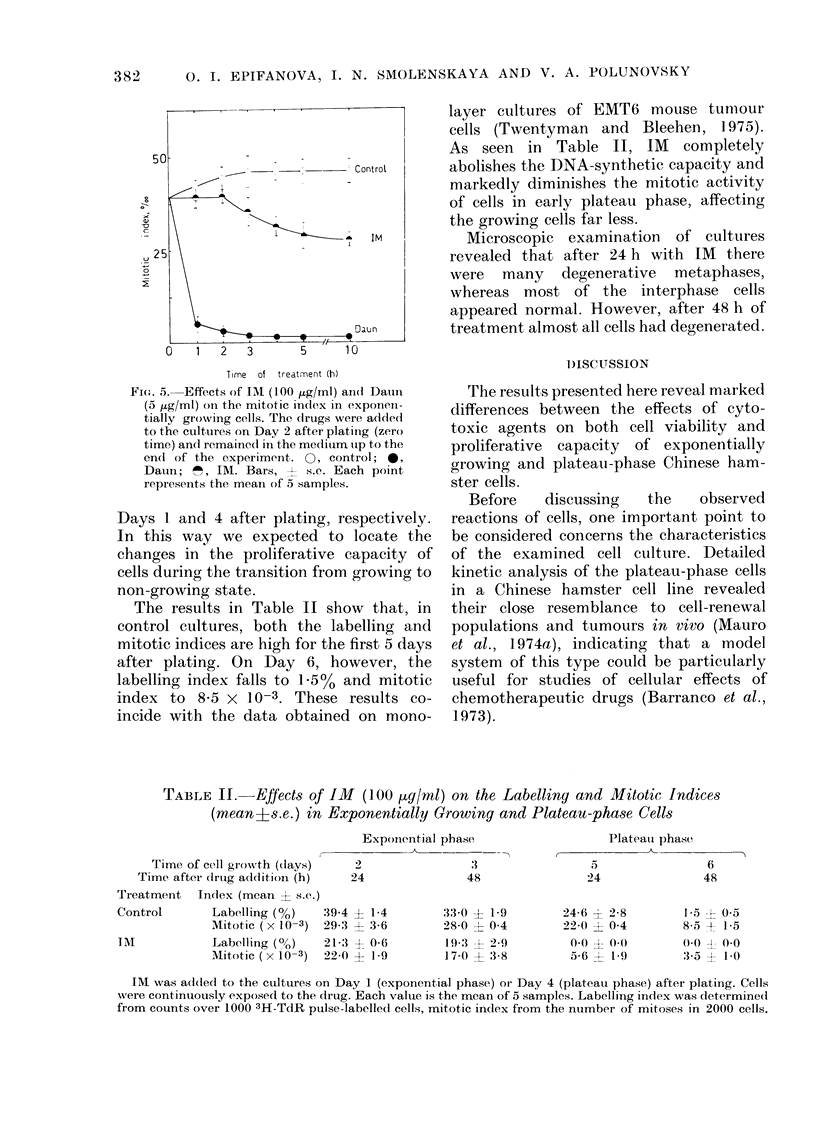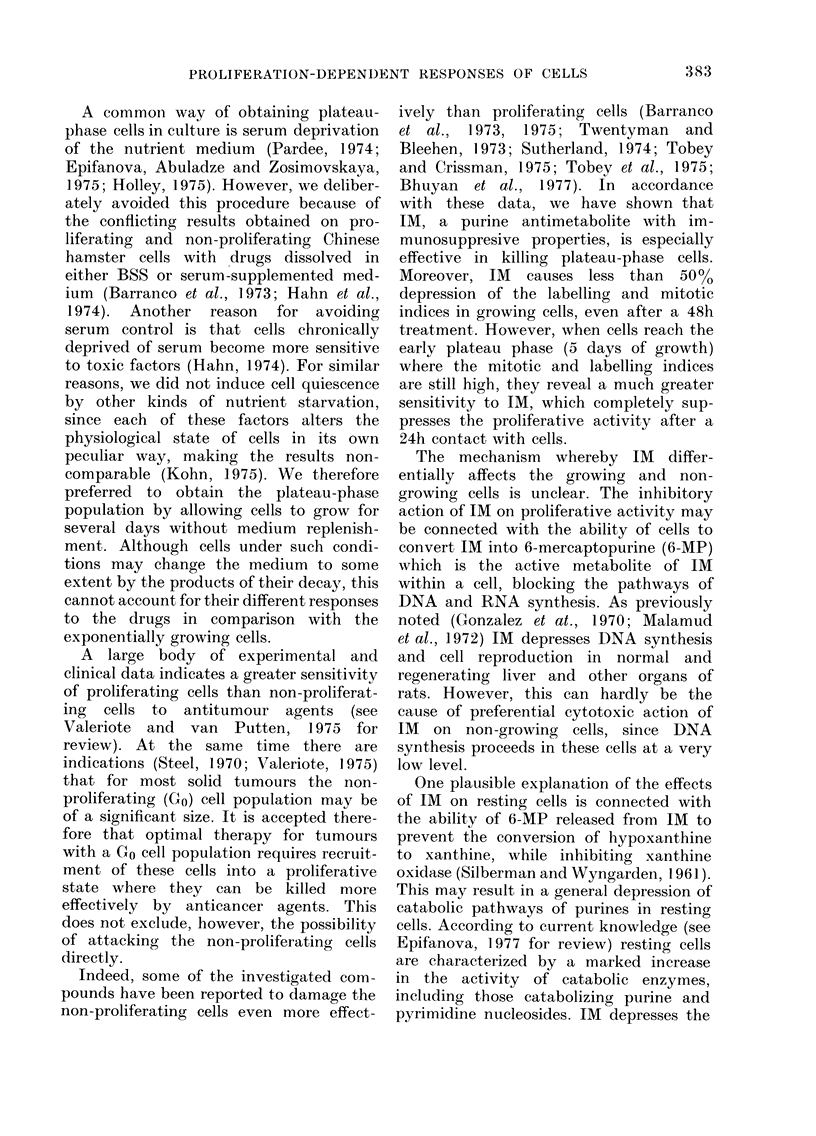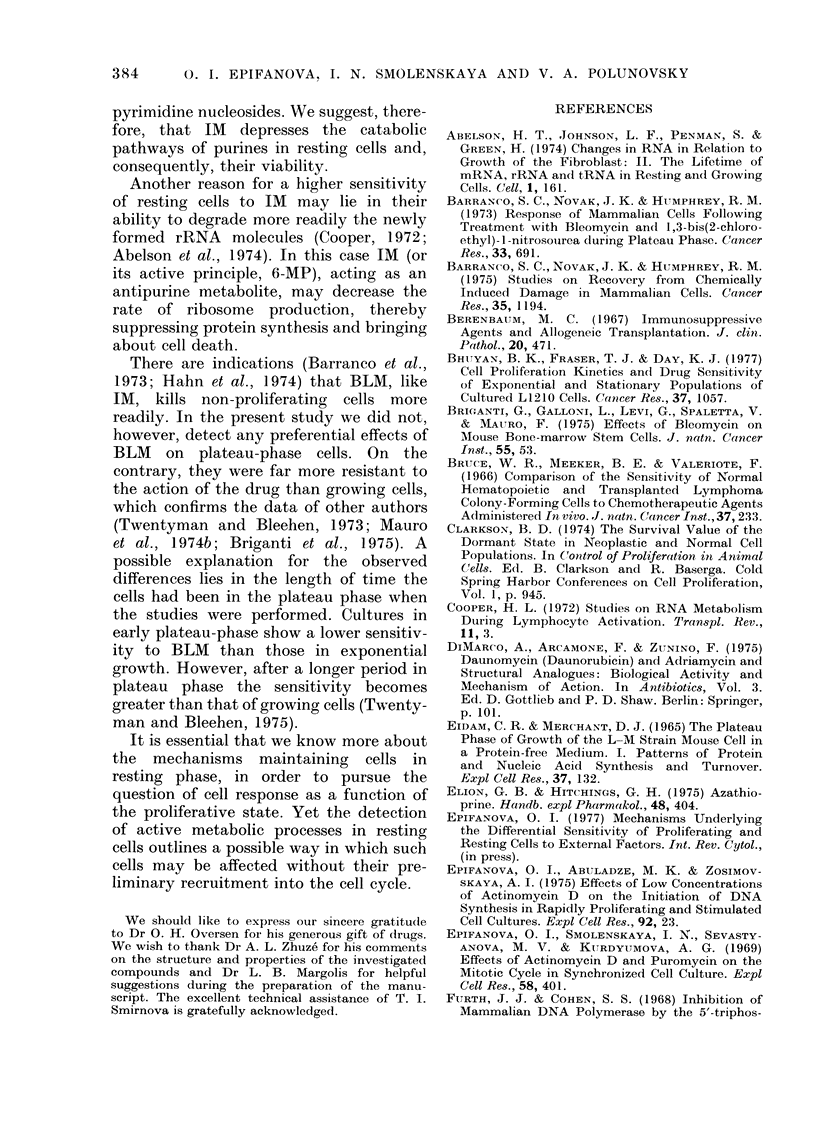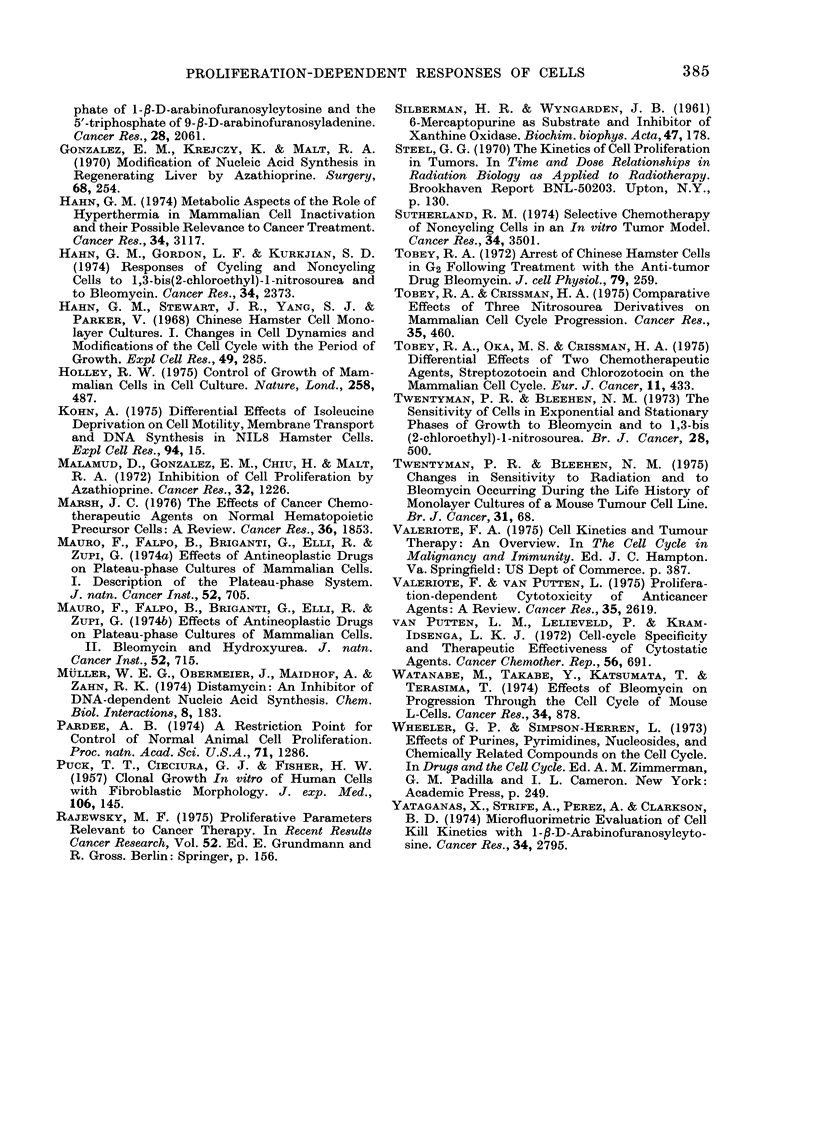# Responses of proliferating and non-proliferating Chinese hamster cells to cytotoxic agents.

**DOI:** 10.1038/bjc.1978.57

**Published:** 1978-03

**Authors:** O. I. Epifanova, I. N. Smolenskaya, V. A. Polunovsky

## Abstract

The effects of various cytotoxic chemicals, as measured by viable cell counts, colony-forming ability and proliferative capacity, have been studied using Chinese hamster cells in exponential and plateau (stationary) phases of growth. The proliferating cells were altogether more sensitive to the action of the drugs than non-proliferating cells. However, imuran (azathioprine) a purine antimetabolite, was more effective against the plateau-phase cells. The observed response of cells to imuran could be detected at a wide range of concentrations (1-100 microgram/ml). These findings are discussed in view of the possible ability of imuran to interfere with active metabolic processes in non-proliferating cells.


*Br. J. Cancer* (1978) **37**, 377

### RESPONSES OF PROLIFERATING AND NON-PROLIFERATING CHINESE HAMSTER CELLS TO CYTOTOXIC AGENTS

O. I. EPIFANOVA, I. N. SMOLENSKAYA AND V. A. POLUNOVSKY

*From the Laboratory for the Functional Morphology of Chromosomes, Institute of Molecular Biology, U.S.S.R. Academy of Sciences, Vavilov St. 32, Moscow B-312, U.S.S.R.*

Received 3 May 1977 Accepted 12 October 1977

**Summary.—The effects of various cytotoxic chemicals, as measured by viable cell counts, colony-forming ability and proliferative capacity, have been studied using Chinese hamster cells in exponential and plateau (stationary) phases of growth. The proliferating cells were altogether more sensitive to the action of the drugs than non-proliferating cells. However, imuran (azathioprine) a purine antimetabolite, was more effective against the plateau-phase cells. The observed response of cells to imuran could be detected at a wide range of concentrations (1–100 μg/ml). These findings are discussed in view of the possible ability of imuran to interfere with active metabolic processes in non-proliferating cells.**

THE main aim of the present investigation was to gain insight into the relationship between the cellular effects of antineoplastic agents and the proliferative state of the exposed cell population.

There is a general agreement (Wheeler and Simpson-Herren, 1973; Clarkson, 1974; Valeriote and van Putten, 1975) that the faster the growth of a cell population, the greater is the susceptibility of that population to the cytocidal effects of antimetabolites. Indeed, numerous experimental data obtained on various cell systems definitely show that proliferating cells are altogether more sensitive to cytotoxic agents than resting cells (Bruce, Meeker and Valeriote, 1966; van Putten, Lelieveld and Kram-Idsenga, 1972; Rajewsky, 1975). Hence, the main way of destroying non-proliferating cells, according to the current schedules (Valeriote, 1975; Valeriote and van Putten, 1975) consists in their recruitment into the cell cycle to make them targets for the appropriate inhibitors.

However, in the course of recent years, evidence has been collected that, in some situations, the non-proliferating cells may reveal an even greater sensitivity to drugs than proliferating cells (Barranco, Novak and Humphrey, 1973, 1975; Twentyman and Bleehen, 1973; Hahn, Gordon and Kurkjian, 1974; Sutherland, 1974; Tobey and Crissman, 1975; Tobey, Oka and Crissman, 1975; see also Marsh, 1976, for review). Although some of the results appear to be conflicting, there is no doubt at present that, at least under certain conditions, the non-proliferating cells may be severely damaged by concentrations of drugs less toxic to the proliferating cells. In this respect, the search for cytotoxic agents directly affecting the non-proliferating cells may be of great importance for cancer therapy.

The studies reported here are concerned with the sensitivity of proliferating and non-proliferating cells to cytotoxic agents of different origin. The agents used were 1-β-D-arabinofuranosylcytosine (ara-C), bleomycin (BLM), daunorubicin (Daun), distamycin A (DST-A) and imuran (IM). Ara-C is an antimetabolite that kills cells by interfering with DNA synthesis through inhibition of DNA polymerase (Furth and Cohen, 1968). BLM is a glycopeptide antibiotic arresting cells in the $G_2$ period of the cell cycle (Tobey, 1972; Watanabe

*et al.*, 1974). Daun, an antibiotic of the anthracycline group, has been shown to intercalate into DNA, thus inhibiting both RNA and DNA synthesis (Di Marco, Arcamone and Zunino, 1975). DST-A, a basic oligopeptide antibiotic, exerts the same effect, acting as an inhibitor of DNA-dependent nucleic-acid synthesis (Müller *et al.*, 1974). IM is a purine antimetabolite used as an immunosuppressive agent (Berenbaum, 1967; Elion and Hitchings, 1975).

### MATERIALS AND METHODS

*Cells and cell-culture technique.*—Chinese hamster cells (Strain Blld-ii-FAF 28, Clone 431) grown as monolayers were used. Stock cultures were maintained in glass bottles in Eagle nutrient medium supplemented with 10% bovine serum, 100 u penicillin, and 50 u streptomycin. Cells were shown to be free of PPLO by fluorescent microscopy after staining with acridine orange.

*Cell counts and survival determinations.*—To study the effects of drugs on cell viability, cultures were seeded into 15ml Leighton tubes without coverslips, in 3 ml of medium and allowed to grow as monolayers. Cells were trypsinized thereafter (0·25% for 2 min) and repeatedly pipetted to disperse clumps. At designated times, aliquots of cell suspension from 5 tubes were counted in a haemocytometer chamber. Ten chambers were used for each of 5 samples. The counts were corrected for dead cells by microscopic examination of 200 cells per sample in the presence of 0·1% eosin solution. Viable cells were defined as cells that were neither stained nor severely distorted morphologically. This technique allows one to determine how many cells are actually killed during continuous drug exposure.

Survival was determined by the ability of cells to form colonies (Puck, Cieciura and Fisher, 1957). This technique has been used in the present study to check the reproductive capacity of surviving cells following brief drug treatment. For these experiments, known numbers of single cells were plated into 60mm Petri dishes ($\sim$ 500 cells per dish) and incubated at 37°C in an atmosphere of 95% air: 5% $CO_2$ for 7 to 9 days. The pH was measured in the course of the experiments and maintained between 7·2 and 7·4. The colonies were stained with 3% methylene blue and counted. Five replicate plates were used for each survival determination.

*Autoradiography and mitotic counts.*—For these investigations, cultures were seeded into 15ml Leighton tubes on coverslips. To obtain autoradiographs, cells were pulsed with $^3$H-thymidine ($^3$H-TdR) at a concentration of 1 $\mu$Ci/ml (sp. act. 5 Ci/mmol) for 30 min before fixing. The coverslips were fixed in acetic acid–ethanol (1:3) and autoradiographs were obtained using liquid emulsion of M type (NiiChimPhoto, Moscow) as described previously (Epifanova *et al.*, 1969). The labelling index was determined by counting the $^3$H-TdR-labelled cells per 1000 cells. Mitotic index was determined from counting the number of mitoses per 2000 cells in fixed preparations stained with Mayer's haematoxylin. Five samples were taken for each fixation.

*Preparation of cells in exponential and plateau (stationary) phases of growth.*—Exponentially growing cultures were obtained by seeding 6 $\times$ 10$^4$ cells per ml. After 48 h of growth, cells were used in experimental studies. The average population-doubling time in exponential growth was 16–18 h. To obtain plateau-phase cultures, cells were allowed to grow further without medium replenishment. Cultures were considered to be in the early plateau phase by the 5th–6th days of growth, when they reached a cell density of $\sim$1·5 $\times$ 10$^5$/cm$^2$, and at the terminal stage of this phase by the 10th day of growth, when a decrease in cell viability was observed (Eidam and Merchant, 1965; Hahn *et al.*, 1968).

*Drug treatment.*—Ara-C (Cytosar) was purchased from the Upjohn s.a., Puurs, Belgium; BLM was kindly supplied by H. Lundbeck and Co. A/S, Copenhagen; Daun (Cerubidin) was obtained from Pharma Rhodia, Birkerod, Denmark; DST-A from Farmitalia Research Labs, Milan; and IM (azathioprine sodium) from Burroughs Wellcome and Co., London. Drug solutions were prepared immediately before use to ensure against loss of activity. All drugs were dissolved in Hanks' balanced salt solution (HBSS) and then diluted to final concentrations. The drugs were used at concentrations exhibiting an optimal effect in cell-killing studies on cultured Chinese hamster cells (Barranco *et al.*, 1973, 1975; Tobey, 1972; Yataganas *et al.*, 1974). The time and duration of treatment varied, depending upon the type of the experiment. The details will be given in the following section of the paper as required.

### RESULTS

A typical growth curve obtained in the present study is shown in Fig. 1. It presents an initial lag phase, in which cells are being attached to the glass surface, followed by an exponential or log phase, and finally by a stationary or plateau phase, in which cell numbers reach a maximum level of $5 \times 10^5$ cells per ml of culture medium ($\sim 1 \cdot 5 \times 10^5/cm^2$). The drugs were usually added to the cultures on the 2nd day after seeding (to study the sensitivity of exponentially growing cells) and on the 6th day after seeding (to study the sensitivity of plateau-phase cells) unless otherwise indicated. During exponential growth, cell viability (judged by staining) generally exceeded 90%. The plateau-phase cultures maintained a high viability (about 75%) up to the 9th day of growth, which allowed us to investigate the response of cells to prolonged drug treatment.

FIG. 1.—Change in cell number with time after seeding $6 \times 10^4$ cells/ml Chinese hamster cells in normal culture. Arrows indicate the points at which the drugs were added. See text for explanation.

FIG. 2.—Effects of various cytotoxic agents on number of viable cells in exponentially growing (□) and plateau-phase (▨) cells after 72 h of treatment. The drugs were added to the cultures on Day 2 (exponential phase) or on Day 6 (plateau phase) after plating. Cell counts were made as described in Materials and Methods. The data are presented as percentage inhibition of viable cells calculated as follows:

$$\frac{\text{Number of viable cells in control culture} - \text{Number of viable cells in treated culture}}{\text{Number of viable cells in control culture}} \times 100\%$$

Bars, ± s.e.

The diagram in Fig. 2 represents the results of a viable cell-counting experiment where cells were continuously incubated with drugs for 72 h, either in exponential or in the plateau phase of growth. It can be seen that growing cells (white columns) are more sensitive to the action of cytotoxic agents than non-growing cells (shaded columns). Among the examined drugs, the greatest general cytocidal effect is shown by Daun, which, under given conditions, causes a 100% reduction in number of viable cells in growing cultures and about 40% in plateau-phase cultures. In this experiment, our attention was attracted to IM, which, contrary to other drugs tested, appeared to be more effective in killing non-proliferating than proliferating cells.

We therefore made a cell-counting experiment to obtain data on the viability of exponentially growing and plateau-

TABLE I.—*Effects of IM* (100 *g/ml*) *and Daun* (5 *μg/ml*) *on Total Numbers of Cells and* % *Dead Cells in Exponentially Growing and Plateau-phase Cells*

| | Exponential phase | | | | Plateau phase | | | |
|---|---|---|---|---|---|---|---|---|
| Time of cell growth (days) | 3 | | 4 | | 7 | | 8 | |
| Time after drug addition (h) | 24 | | 48 | | 24 | | 48 | |
| | Cell parameters (mean ± s.e.) | | | | | | | |
| Treatment | N | D (%) | N | D (%) | N | D (%) | N | D (%) |
| Control | 7·6 ± 0·6 | 4·5 ± 0·2 | 16·0 ± 6·4 | 7·0 ± 0·5 | 22·3 ± 5·2 | 8·0 ± 0·4 | 20·8 ± 9·8 | 9·2 ± 0·4 |
| IM | 3·0 ± 0·3 | 12·5 ± 0·7 | 2·3 ± 0·3 | 21·3 ± 1·8 | 7·7 ± 3·0 | 56·2 ± 2·4 | 1·1 ± 0·6 | 87·9 ± 0·9 |
| Daun | 1·2 ± 0·3 | 46·2 ± 9·1 | 0·8 ± 0·05 | 92·3 ± 0·2 | 15·0 ± 9·0 | 27·9 ± 5·0 | 4·5 ± 1·7 | 58·9 ± 0·1 |

N, number of cells (per ml × $10^4$); D, % of dead (eosin stained) cells. Drugs were added to the cultures on Day 2 (exponential phase) or on Day 6 (plateau phase) after plating. Cells were counted as in Materials and Methods. The results represent the mean of 4 experiments.

FIG. 3.—Viability of exponentially growing (●) and plateau-phase (○) cells exposed to graded concentrations of IM after 48 h of treatment. IM was added to the cultures on Day 2 (exponential phase) or on Day 6 (plateau phase) after plating. Cell counts were made as described in Materials and Methods. Bars, ± s.e.

FIG. 4.—Effects of IM (100 μg/ml) and Daun (5 μg/ml) on the colony-forming ability of exponentially growing (A) and plateau phase (B) cells. C, control; D, Daun; IM, imuran. Cells were treated with drugs for 1 h in either exponential or plateau phase (on Days 2 and 6 of growth, respectively) and plated into Petri dishes immediately after treatment. Bars, ± s.e. The results represent the average of 3 experiments.

phase cells exposed to graded concentrations of IM. The results of this experiment are given in Fig. 3. Again we can see that non-proliferating cells are more vulnerable to IM than proliferating cells, being affected even at concentrations as low as 1·0 and 10 μg/ml, (*i.e.* 10- and 100-fold smaller than that initially tested).

To investigate the question further, we compared the effects of IM on cell viability in the exponential and plateau phases of growth, performing the cell counts after a 24- and 48h drug exposure. The data in Table I summarize the results of 4 experiments which confirm and extend the previously obtained results showing that IM kills the non-proliferating cells more readily than the growing cells. The observed differences are very pronounced from the beginning of the experiment. In Table I are also shown the data obtained with Daun which, under similar conditions, exerts a preferential effect on growing cells.

In the following experiment, we studied the effects of IM and Daun on the colony-forming ability of cells treated for 1 h in either exponential or plateau-phase growth. The results in Fig. 4 show that cells treated with IM in the exponential phase (A) reveal a greater survival than those treated in plateau phase (B). In the same situation, Daun affects more readily the colony-forming ability of exponentially growing cells.

The next step was to investigate the effects of IM on the proliferative capacity of growing and non-growing cells. The first experiment on these lines was designed to examine how IM would affect the mitotic activity of a growing cell population during early hours of treatment. The results in Fig. 5 show that IM causes only a 15% depression of the mitotic index, even after a 10h contact with cells. In similar conditions, Daun completely inhibited the mitotic activity of growing cells by the 3rd hour of treatment.

In the following experiment we prolonged the exposure of cells to IM up to 24 and 48 h and compared its effects on the proliferative activity in growing and plateau-phase cultures. This time we started the treatment of cells with IM on

FIG. 5.—Effects of IM (100 μg/ml) and Daun (5 μg/ml) on the mitotic index in exponentially growing cells. The drugs were added to the cultures on Day 2 after plating (zero time) and remained in the medium up to the end of the experiment. ○, control; ●, Daun; ◒, IM. Bars, ± s.e. Each point represents the mean of 5 samples.

Days 1 and 4 after plating, respectively. In this way we expected to locate the changes in the proliferative capacity of cells during the transition from growing to non-growing state.

The results in Table II show that, in control cultures, both the labelling and mitotic indices are high for the first 5 days after plating. On Day 6, however, the labelling index falls to 1·5% and mitotic index to $8{\cdot}5 \times 10^{-3}$. These results coincide with the data obtained on monolayer cultures of EMT6 mouse tumour cells (Twentyman and Bleehen, 1975). As seen in Table II, IM completely abolishes the DNA-synthetic capacity and markedly diminishes the mitotic activity of cells in early plateau phase, affecting the growing cells far less.

Microscopic examination of cultures revealed that after 24 h with IM there were many degenerative metaphases, whereas most of the interphase cells appeared normal. However, after 48 h of treatment almost all cells had degenerated.

### DISCUSSION

The results presented here reveal marked differences between the effects of cytotoxic agents on both cell viability and proliferative capacity of exponentially growing and plateau-phase Chinese hamster cells.

Before discussing the observed reactions of cells, one important point to be considered concerns the characteristics of the examined cell culture. Detailed kinetic analysis of the plateau-phase cells in a Chinese hamster cell line revealed their close resemblance to cell-renewal populations and tumours *in vivo* (Mauro *et al.*, 1974*a*), indicating that a model system of this type could be particularly useful for studies of cellular effects of chemotherapeutic drugs (Barranco *et al.*, 1973).

TABLE II.—*Effects of IM* (100 *μg/ml*) *on the Labelling and Mitotic Indices* (*mean±s.e.*) *in Exponentially Growing and Plateau-phase Cells*

| | | Exponential phase | | Plateau phase | |
|---|---|---|---|---|---|
| Time of cell growth (days) | | 2 | 3 | 5 | 6 |
| Time after drug addition (h) | | 24 | 48 | 24 | 48 |
| Treatment | Index (mean ± s.e.) | | | | |
| Control | Labelling (%) | 39·4 ± 1·4 | 33·0 ± 1·9 | 24·6 ± 2·8 | 1·5 ± 0·5 |
| | Mitotic ($\times 10^{-3}$) | 29·3 ± 3·6 | 28·0 ± 0·4 | 22·0 ± 0·4 | 8·5 ± 1·5 |
| IM | Labelling (%) | 21·3 ± 0·6 | 19·3 ± 2·9 | 0·0 ± 0·0 | 0·0 ± 0·0 |
| | Mitotic ($\times 10^{-3}$) | 22·0 ± 1·9 | 17·0 ± 3·8 | 5·6 ± 1·9 | 3·5 ± 1·0 |

IM was added to the cultures on Day 1 (exponential phase) or Day 4 (plateau phase) after plating. Cells were continuously exposed to the drug. Each value is the mean of 5 samples. Labelling index was determined from counts over 1000 $^3$H-TdR pulse-labelled cells, mitotic index from the number of mitoses in 2000 cells.

A common way of obtaining plateau-phase cells in culture is serum deprivation of the nutrient medium (Pardee, 1974; Epifanova, Abuladze and Zosimovskaya, 1975; Holley, 1975). However, we deliberately avoided this procedure because of the conflicting results obtained on proliferating and non-proliferating Chinese hamster cells with drugs dissolved in either BSS or serum-supplemented medium (Barranco *et al.*, 1973; Hahn *et al.*, 1974). Another reason for avoiding serum control is that cells chronically deprived of serum become more sensitive to toxic factors (Hahn, 1974). For similar reasons, we did not induce cell quiescence by other kinds of nutrient starvation, since each of these factors alters the physiological state of cells in its own peculiar way, making the results non-comparable (Kohn, 1975). We therefore preferred to obtain the plateau-phase population by allowing cells to grow for several days without medium replenishment. Although cells under such conditions may change the medium to some extent by the products of their decay, this cannot account for their different responses to the drugs in comparison with the exponentially growing cells.

A large body of experimental and clinical data indicates a greater sensitivity of proliferating cells than non-proliferating cells to antitumour agents (see Valeriote and van Putten, 1975 for review). At the same time there are indications (Steel, 1970; Valeriote, 1975) that for most solid tumours the non-proliferating ($G_0$) cell population may be of a significant size. It is accepted therefore that optimal therapy for tumours with a $G_0$ cell population requires recruitment of these cells into a proliferative state where they can be killed more effectively by anticancer agents. This does not exclude, however, the possibility of attacking the non-proliferating cells directly.

Indeed, some of the investigated compounds have been reported to damage the non-proliferating cells even more effectively than proliferating cells (Barranco *et al.*, 1973, 1975; Twentyman and Bleehen, 1973; Sutherland, 1974; Tobey and Crissman, 1975; Tobey *et al.*, 1975; Bhuyan *et al.*, 1977). In accordance with these data, we have shown that IM, a purine antimetabolite with immunosuppresive properties, is especially effective in killing plateau-phase cells. Moreover, IM causes less than 50% depression of the labelling and mitotic indices in growing cells, even after a 48h treatment. However, when cells reach the early plateau phase (5 days of growth) where the mitotic and labelling indices are still high, they reveal a much greater sensitivity to IM, which completely suppresses the proliferative activity after a 24h contact with cells.

The mechanism whereby IM differentially affects the growing and non-growing cells is unclear. The inhibitory action of IM on proliferative activity may be connected with the ability of cells to convert IM into 6-mercaptopurine (6-MP) which is the active metabolite of IM within a cell, blocking the pathways of DNA and RNA synthesis. As previously noted (Gonzalez *et at.*, 1970; Malamud *et al.*, 1972) IM depresses DNA synthesis and cell reproduction in normal and regenerating liver and other organs of rats. However, this can hardly be the cause of preferential cytotoxic action of IM on non-growing cells, since DNA synthesis proceeds in these cells at a very low level.

One plausible explanation of the effects of IM on resting cells is connected with the ability of 6-MP released from IM to prevent the conversion of hypoxanthine to xanthine, while inhibiting xanthine oxidase (Silberman and Wyngarden, 1961). This may result in a general depression of catabolic pathways of purines in resting cells. According to current knowledge (see Epifanova, 1977 for review) resting cells are characterized by a marked increase in the activity of catabolic enzymes, including those catabolizing purine and pyrimidine nucleosides. IM depresses the pyrimidine nucleosides. We suggest, therefore, that IM depresses the catabolic pathways of purines in resting cells and, consequently, their viability.

Another reason for a higher sensitivity of resting cells to IM may lie in their ability to degrade more readily the newly formed rRNA molecules (Cooper, 1972; Abelson *et al.*, 1974). In this case IM (or its active principle, 6-MP), acting as an antipurine metabolite, may decrease the rate of ribosome production, thereby suppressing protein synthesis and bringing about cell death.

There are indications (Barranco *et al.*, 1973; Hahn *et al.*, 1974) that BLM, like IM, kills non-proliferating cells more readily. In the present study we did not, however, detect any preferential effects of BLM on plateau-phase cells. On the contrary, they were far more resistant to the action of the drug than growing cells, which confirms the data of other authors (Twentyman and Bleehen, 1973; Mauro *et al.*, 1974*b*; Briganti *et al.*, 1975). A possible explanation for the observed differences lies in the length of time the cells had been in the plateau phase when the studies were performed. Cultures in early plateau-phase show a lower sensitivity to BLM than those in exponential growth. However, after a longer period in plateau phase the sensitivity becomes greater than that of growing cells (Twentyman and Bleehen, 1975).

It is essential that we know more about the mechanisms maintaining cells in resting phase, in order to pursue the question of cell response as a function of the proliferative state. Yet the detection of active metabolic processes in resting cells outlines a possible way in which such cells may be affected without their preliminary recruitment into the cell cycle.

We should like to express our sincere gratitude to Dr O. H. Oversen for his generous gift of drugs. We wish to thank Dr A. L. Zhuzé for his comments on the structure and properties of the investigated compounds and Dr L. B. Margolis for helpful suggestions during the preparation of the manuscript. The excellent technical assistance of T. I. Smirnova is gratefully acknowledged.

REFERENCES

ABELSON, H. T., JOHNSON, L. F., PENMAN, S. & GREEN, H. (1974) Changes in RNA in Relation to Growth of the Fibroblast: II. The Lifetime of mRNA, rRNA and tRNA in Resting and Growing Cells. *Cell*, **1**, 161.

BARRANCO, S. C., NOVAK, J. K. & HUMPHREY, R. M. (1973) Response of Mammalian Cells Following Treatment with Bleomycin and 1,3-bis(2-chloroethyl)-1-nitrosourea during Plateau Phase. *Cancer Res.*, **33**, 691.

BARRANCO, S. C., NOVAK, J. K. & HUMPHREY, R. M. (1975) Studies on Recovery from Chemically Induced Damage in Mammalian Cells. *Cancer Res.*, **35**, 1194.

BERENBAUM, M. C. (1967) Immunosuppressive Agents and Allogeneic Transplantation. *J. clin. Pathol.*, **20**, 471.

BHUYAN, B. K., FRASER, T. J. & DAY, K. J. (1977) Cell Proliferation Kinetics and Drug Sensitivity of Exponential and Stationary Populations of Cultured L1210 Cells. *Cancer Res.*, **37**, 1057.

BRIGANTI, G., GALLONI, L., LEVI, G., SPALETTA, V. & MAURO, F. (1975) Effects of Bleomycin on Mouse Bone-marrow Stem Cells. *J. natn. Cancer Inst.*, **55**, 53.

BRUCE, W. R., MEEKER, B. E. & VALERIOTE, F. (1966) Comparison of the Sensitivity of Normal Hematopoietic and Transplanted Lymphoma Colony-Forming Cells to Chemotherapeutic Agents Administered *In vivo*. *J. natn. Cancer Inst.*, **37**, 233.

CLARKSON, B. D. (1974) The Survival Value of the Dormant State in Neoplastic and Normal Cell Populations. In *Control of Proliferation in Animal Cells*. Ed. B. Clarkson and R. Baserga. Cold Spring Harbor Conferences on Cell Proliferation, Vol. 1, p. 945.

COOPER, H. L. (1972) Studies on RNA Metabolism During Lymphocyte Activation. *Transpl. Rev.*, **11**, 3.

DIMARCO, A., ARCAMONE, F. & ZUNINO, F. (1975) Daunomycin (Daunorubicin) and Adriamycin and Structural Analogues: Biological Activity and Mechanism of Action. In *Antibiotics*, Vol. 3. Ed. D. Gottlieb and P. D. Shaw. Berlin: Springer, p. 101.

EIDAM, C. R. & MERCHANT, D. J. (1965) The Plateau Phase of Growth of the L–M Strain Mouse Cell in a Protein-free Medium. I. Patterns of Protein and Nucleic Acid Synthesis and Turnover. *Expl Cell Res.*, **37**, 132.

ELION, G. B. & HITCHINGS, G. H. (1975) Azathioprine. *Handb. expl Pharmakol.*, **48**, 404.

EPIFANOVA, O. I. (1977) Mechanisms Underlying the Differential Sensitivity of Proliferating and Resting Cells to External Factors. *Int. Rev. Cytol.*, (in press).

EPIFANOVA, O. I., ABULADZE, M. K. & ZOSIMOVSKAYA, A. I. (1975) Effects of Low Concentrations of Actinomycin D on the Initiation of DNA Synthesis in Rapidly Proliferating and Stimulated Cell Cultures. *Expl Cell Res.*, **92**, 23.

EPIFANOVA, O. I., SMOLENSKAYA, I. N., SEVASTYANOVA, M. V. & KURDYUMOVA, A. G. (1969) Effects of Actinomycin D and Puromycin on the Mitotic Cycle in Synchronized Cell Culture. *Expl Cell Res.*, **58**, 401.

FURTH, J. J. & COHEN, S. S. (1968) Inhibition of Mammalian DNA Polymerase by the 5′-triphosphate of 1-β-D-arabinofuranosylcytosine and the 5′-triphosphate of 9-β-D-arabinofuranosyladenine. *Cancer Res.*, **28**, 2061.

GONZALEZ, E. M., KREJCZY, K. & MALT, R. A. (1970) Modification of Nucleic Acid Synthesis in Regenerating Liver by Azathioprine. *Surgery*, **68**, 254.

HAHN, G. M. (1974) Metabolic Aspects of the Role of Hyperthermia in Mammalian Cell Inactivation and their Possible Relevance to Cancer Treatment. *Cancer Res.*, **34**, 3117.

HAHN, G. M., GORDON, L. F. & KURKJIAN, S. D. (1974) Responses of Cycling and Noncycling Cells to 1,3-bis(2-chloroethyl)-1-nitrosourea and to Bleomycin. *Cancer Res.*, **34**, 2373.

HAHN, G. M., STEWART, J. R., YANG, S. J. & PARKER, V. (1968) Chinese Hamster Cell Monolayer Cultures. I. Changes in Cell Dynamics and Modifications of the Cell Cycle with the Period of Growth. *Expl Cell Res.*, **49**, 285.

HOLLEY, R. W. (1975) Control of Growth of Mammalian Cells in Cell Culture. *Nature, Lond.*, **258**, 487.

KOHN, A. (1975) Differential Effects of Isoleucine Deprivation on Cell Motility, Membrane Transport and DNA Synthesis in NIL8 Hamster Cells. *Expl Cell Res.*, **94**, 15.

MALAMUD, D., GONZALEZ, E. M., CHIU, H. & MALT, R. A. (1972) Inhibition of Cell Proliferation by Azathioprine. *Cancer Res.*, **32**, 1226.

MARSH, J. C. (1976) The Effects of Cancer Chemotherapeutic Agents on Normal Hematopoietic Precursor Cells: A Review. *Cancer Res.*, **36**, 1853.

MAURO, F., FALPO, B., BRIGANTI, G., ELLI, R. & ZUPI, G. (1974*a*) Effects of Antineoplastic Drugs on Plateau-phase Cultures of Mammalian Cells. I. Description of the Plateau-phase System. *J. natn. Cancer Inst.*, **52**, 705.

MAURO, F., FALPO, B., BRIGANTI, G., ELLI, R. & ZUPI, G. (1974*b*) Effects of Antineoplastic Drugs on Plateau-phase Cultures of Mammalian Cells. II. Bleomycin and Hydroxyurea. *J. natn. Cancer Inst.*, **52**, 715.

MÜLLER, W. E. G., OBERMEIER, J., MAIDHOF, A. & ZAHN, R. K. (1974) Distamycin: An Inhibitor of DNA-dependent Nucleic Acid Synthesis. *Chem. Biol. Interactions*, **8**, 183.

PARDEE, A. B. (1974) A Restriction Point for Control of Normal Animal Cell Proliferation. *Proc. natn. Acad. Sci. U.S.A.*, **71**, 1286.

PUCK, T. T., CIECIURA, G. J. & FISHER, H. W. (1957) Clonal Growth *In vitro* of Human Cells with Fibroblastic Morphology. *J. exp. Med.*, **106**, 145.

RAJEWSKY, M. F. (1975) Proliferative Parameters Relevant to Cancer Therapy. In *Recent Results Cancer Research*, Vol. **52**. Ed. E. Grundmann and R. Gross. Berlin: Springer, p. 156.

SILBERMAN, H. R. & WYNGARDEN, J. B. (1961) 6-Mercaptopurine as Substrate and Inhibitor of Xanthine Oxidase. *Biochim. biophys. Acta*, **47**, 178.

STEEL, G. G. (1970) The Kinetics of Cell Proliferation in Tumors. In *Time and Dose Relationships in Radiation Biology as Applied to Radiotherapy*. Brookhaven Report BNL-50203. Upton, N.Y., p. 130.

SUTHERLAND, R. M. (1974) Selective Chemotherapy of Noncycling Cells in an *In vitro* Tumor Model. *Cancer Res.*, **34**, 3501.

TOBEY, R. A. (1972) Arrest of Chinese Hamster Cells in $G_2$ Following Treatment with the Anti-tumor Drug Bleomycin. *J. cell Physiol.*, **79**, 259.

TOBEY, R. A. & CRISSMAN, H. A. (1975) Comparative Effects of Three Nitrosourea Derivatives on Mammalian Cell Cycle Progression. *Cancer Res.*, **35**, 460.

TOBEY, R. A., OKA, M. S. & CRISSMAN, H. A. (1975) Differential Effects of Two Chemotherapeutic Agents, Streptozotocin and Chlorozotocin on the Mammalian Cell Cycle. *Eur. J. Cancer*, **11**, 433.

TWENTYMAN, P. R. & BLEEHEN, N. M. (1973) The Sensitivity of Cells in Exponential and Stationary Phases of Growth to Bleomycin and to 1,3-bis (2-chloroethyl)-1-nitrosourea. *Br. J. Cancer*, **28**, 500.

TWENTYMAN, P. R. & BLEEHEN, N. M. (1975) Changes in Sensitivity to Radiation and to Bleomycin Occurring During the Life History of Monolayer Cultures of a Mouse Tumour Cell Line. *Br. J. Cancer*, **31**, 68.

VALERIOTE, F. A. (1975) Cell Kinetics and Tumour Therapy: An Overview. In *The Cell Cycle in Malignancy and Immunity*. Ed. J. C. Hampton. Va. Springfield: US Dept of Commerce. p. 387.

VALERIOTE, F. & VAN PUTTEN, L. (1975) Proliferation-dependent Cytotoxicity of Anticancer Agents: A Review. *Cancer Res.*, **35**, 2619.

VAN PUTTEN, L. M., LELIEVELD, P. & KRAM-IDSENGA, L. K. J. (1972) Cell-cycle Specificity and Therapeutic Effectiveness of Cytostatic Agents. *Cancer Chemother. Rep.*, **56**, 691.

WATANABE, M., TAKABE, Y., KATSUMATA, T. & TERASIMA, T. (1974) Effects of Bleomycin on Progression Through the Cell Cycle of Mouse L-Cells. *Cancer Res.*, **34**, 878.

WHEELER, G. P. & SIMPSON-HERREN, L. (1973) Effects of Purines, Pyrimidines, Nucleosides, and Chemically Related Compounds on the Cell Cycle. In *Drugs and the Cell Cycle*. Ed. A. M. Zimmerman, G. M. Padilla and I. L. Cameron. New York: Academic Press, p. 249.

YATAGANAS, X., STRIFE, A., PEREZ, A. & CLARKSON, B. D. (1974) Microfluorimetric Evaluation of Cell Kill Kinetics with 1-β-D-Arabinofuranosylcytosine. *Cancer Res.*, **34**, 2795.